# Pluripotency and immunomodulatory signatures of canine induced pluripotent stem cell-derived mesenchymal stromal cells are similar to harvested mesenchymal stromal cells

**DOI:** 10.1038/s41598-021-82856-3

**Published:** 2021-02-10

**Authors:** Arash Shahsavari, Prasanna Weeratunga, Dmitry A. Ovchinnikov, Deanne J. Whitworth

**Affiliations:** 1 School of Veterinary Science, University of Queensland, Gatton, QLD 4343 Australia; 2 Australian Institute for Bioengineering and Nanotechnology, University of Queensland, St Lucia, QLD 4067 Australia

**Keywords:** Mesenchymal stem cells, Induced pluripotent stem cells

## Abstract

With a view towards harnessing the therapeutic potential of canine mesenchymal stromal cells (cMSCs) as modulators of inflammation and the immune response, and to avoid the issues of the variable quality and quantity of harvested cMSCs, we examined the immunomodulatory properties of cMSCs derived from canine induced pluripotent stem cells (ciMSCs), and compared them to cMSCs harvested from adipose tissue (cAT-MSC) and bone marrow (cBM-MSC). A combination of deep sequencing and quantitative RT-PCR of the ciMSC transcriptome confirmed that ciMSCs express more genes in common with cBM-MSCs and cAT-MSCs than with the ciPSCs from which they were derived. Both ciMSCs and harvested cMSCs express a range of pluripotency factors in common with the ciPSCs including *NANOG, POU5F1 (OCT-4), SOX-2, KLF-4, LIN-28A, MYC, LIF, LIFR*, and *TERT*. However, *ESRRB* and *PRDM-14*, both factors associated with naïve, rather than primed, pluripotency were expressed only in the ciPSCs. *CXCR-4,* which is essential for the homing of MSCs to sites of inflammation, is also detectable in ciMSCs, cAT- and cBM-MSCs, but not ciPSCs. ciMSCs constitutively express the immunomodulatory factors *iNOS*, *GAL-9*, *TGF-β1, PTGER-2α* and *VEGF*, and the pro-inflammatory mediators *COX-2, IL-1β* and *IL-8.* When stimulated with the canine pro-inflammatory cytokines tumor necrosis factor-α (cTNF-α), interferon-γ (cIFN-γ), or a combination of both, ciMSCs upregulated their expression of *IDO, iNOS*, *GAL-9, HGF, TGF-β1, PTGER-2α, VEGF, COX-2, IL-1β* and *IL-8.* When co-cultured with mitogen-stimulated lymphocytes, ciMSCs downregulated their expression of *iNOS*, *HGF*, *TGF-β1 *and *PTGER-2α,* while increasing their expression of *COX-2, IDO* and *IL-1β*. Taken together, these findings suggest that ciMSCs possess similar immunomodulatory capabilities as harvested cMSCs and support further investigation into their potential use for the management of canine immune-mediated and inflammatory disorders.

## Introduction

Mesenchymal stromal cells (MSCs), also called mesenchymal stem cells, are multipotent, self-renewing, non-hematopoietic stromal cells that are capable of differentiating into mesenchymal lineages including adipose tissue, bone, cartilage and muscle^1–3^. MSCs are commonly isolated from adipose tissue and bone marrow, but are also found in other tissues such as umbilical cord blood, placenta, skeletal muscle, synovial membranes, nervous tissue and dental pulp^4–6^. MSCs have acquired substantial clinical appeal owing to their immunomodulatory and anti-inflammatory properties^7–11^. The immunosuppressive functions of MSCs are triggered by the tissue microenvironment where pro-inflammatory cytokines such as interferon-γ (IFN-γ), tumor necrosis factor-α (TNF-α), interleukin-1α (IL-1α) and interleukin-1β (IL-1β) are released from activated T cells^12–15^. In humans, MSCs derived from a variety of tissues, including adipose tissue, bone marrow and gingiva have been shown to inhibit the proliferation of CD4 + and CD8 +  T cells, B cells and dendritic cells, and the maturation and activation of natural killer cells^16–19^. The MSC-derived soluble factors responsible for their immunomodulatory effects include indoleamine 2,3 dioxygenase (IDO), induced nitric oxide (iNOS), cyclooxygenase-2 (COX-2), vascular endothelial growth factor (VEGF), interleukin-10 (IL-10), hepatocyte growth factor (HGF), prostaglandin E2 (PGE2), transforming growth factor-β1 (TGF-β1) and haem oxygenase-1 (HO-1)^20–29^. More specifically, the immunosuppressive effect of MSCs is primarily mediated by IDO or iNOS, produced by human and mouse MSCs, respectively, following the stimulatory effect of pro-inflammatory cytokines on MSCs^13,25,30–32^. Canine adipose tissue-derived MSCs (cAT-MSCs) have also been shown to modulate the immune response via the production of iNOS, TGF-β1, HGF, IDO and PGE2, while bone marrow-derived MSCs (cBM-MSCs) secrete TGF-β1 and VEGF^25,33,34^. In the horse, MSCs from bone marrow, adipose tissue, umbilical cord blood and Wharton’s jelly that have been primed with TNF-α and/or IFN-γ similarly inhibit the proliferation of T lymphocytes^35,36^, and analyses specifically on BM-MSCs identified upregulated expression of *IDO*, *iNOS*, *IL-6*, *COX-2* and *VCAM-1*^36–39^. While MSCs can be harvested from a range of tissues, they constitute a very small proportion of the total cells collected: 0.001–0.01% in bone marrow aspirates and 0.05% of cells in adipose tissue^40,41^, posing a challenge in obtaining sufficient cells for therapeutic applications. To overcome this challenge, we have previously generated MSCs from canine induced pluripotent stem cells (ciMSCs) via inhibition of the TGFβ/activin signalling pathway^42^. These ciMSCs express MSC markers and show a comparable differentiation potential to harvested cAT-MSCs and cBM-MSCs, readily forming cartilage, bone and adipose tissue^42^. The current study has expanded the original characterisation of the ciMSCs by comparing their transcriptome with that of harvested cBM-MSCs and of the ciPSCs from which they were derived. Upon confirming that they cluster with the cBM-MSCs and not the ciPSCs, and with the intention of exploring their potential as an ‘off-the-shelf’ MSC-based therapy for controlling immune-mediated and inflammatory diseases in the dog, we further investigated their anti-inflammatory and immunomodulatory profiles in comparison with harvested cAT-MSC and cBM-MSCs.

## Materials and methods

All methods involving the use of animals and/or animal tissues were carried out in accordance with relevant guidelines and regulations. The collection and use of animal tissues was approved by the Animal Ethics Committee at The University of Queensland under ethics approval numbers SVS/194/15, SVS/099/17 and SVS/ANRFA/453/18.

### Culture of ciMSCs, cAT-MSCs and cBM-MSCs

Cultures of ciMSCs^42^, commercially available adult cAT-MSCs (Regeneus Ltd, Australia), and harvested cBM-MSCs^42^ were maintained in MSC-specific medium consisting of KnockOut Dulbecco’s Modified Eagle’s Medium (KnockOut DMEM; Gibco, Thermo Fisher Scientific, Australia) supplemented with 15% (v/v) ESC-qualified fetal bovine serum (HyClone, GE Healthcare Life Sciences, Australia), 0.1 mM Non-Essential Amino Acid solution (NEAA; Gibco), and 2 mM _L_-glutamine (Gibco) at 37 °C with 5% CO_2_.

### RNA isolation, cDNA synthesis and quantitative polymerase chain reaction

Total RNA was extracted using the NucleoSpin RNA kit (Macherey–Nagel GmbH, Thermo Fisher Scientific) and complementary DNA was synthesized using the iScript Reverse Transcriptase kit (Bio-Rad Laboratories, Australia) according to the manufacturer’s instructions. The comparative expression of immunomodulatory and anti-inflammatory factors was performed by real-time quantitative RT-PCR (qRT-PCR) with the SsoFastEva Green Supermix (Bio-Rad) on a CFX-96 real time PCR detection system (Bio-Rad). Data were normalised to the expression level of *cGAPDH*. Validated primers and their product sizes are listed in Supplementary Table 1. The cycling parameters for the qRT-PCR were: denaturation at 95 °C for 3 min, 45 amplification cycles (95 °C, 10 s; 62 °C, 20 s) and elongation at 75 °C for 1 min. Melt curve analysis was performed over a temperature range of 65–95 °C in 0.5 °C increments for 0.05 s. The relative expression ratios of genes were calculated by the Delta Ct method. Dissociation curve analysis was implemented to confirm the specificity of the PCR products.

### Deep sequencing of ciMSC, cBM-MSC and ciPSC transcriptomes

RNA was extracted from one line of each of the ciPSCs (Clone A), ciMSCs (derived from Clone A ciPSCs), and cBM-MSCs as described above. 100 base-pair paired-end mRNA sequencing was performed by the Australian Genome Research Facility Ltd (www.agrf.org.au) on an Illumina HiSeq 4000 platform. Primary sequence data underwent demultiplexing, quality control, alignment, transcript assembly, quantification and normalisation, followed by differential expression analysis, as performed by the AGRF. Sequence reads were screened for the presence of any cross-species contamination and mapped against the canine reference genome CanFam3.1 (GCA_000002285.2) (https://asia.ensembl.org/Canis_familiaris). Genes were defined as expressed if the CPM ≥ 1. EdgeR was used to generate multidimensional scaling (MDS) plots using both raw gene counts and after normalisation by EdgeR’s TMM algorithm to account for the different library sizes for each sample. Both the raw gene count and normalised gene count MDS plots were generated from the data of the 500 most variably expressed genes across all samples. Venn analysis was performed using the Venny tool at http://bioinfogp.cnb.csic.es/tools/venny. Due to financial and logistical constraints only one sample of the ciPSCs and ciMSCs, and cBM-MSCs from one individual, were used for RNA sequencing; therefore, the RNAseq data is indicative of genes that are expressed, but without the number of samples required to perform statistical analyses no comment can be made regarding differential expression between the cell types.

### In vitro stimulation of MSCs with pro-inflammatory cytokines

cAT-MSCs, cBM-MSCs and ciMSCs were plated separately, in duplicate, at a density of 2 × 10^5^ cells/ml with 1 ml of MSC medium (as above) in flat-bottom 24-well cell culture plates (Costar, Corning Life Sciences, Australia). Each of the cAT-MSCs, cBM-MSCs and ciMSCs were cultured with either canine tumor necrosis factor-α (cTNF-α) (10 ng/ml) (VWR International, Australia), canine interferon-γ (cIFN-γ) (200 ng/ml) (VWR International), or both, at 37 °C and 5% CO_2_ for 48 h.

### Isolation of leukocytes from canine blood

40 ml of whole blood was aseptically collected in Vacuette blood collection tubes (InterPath Services, Australia) from two healthy adult mixed-breed dogs at the School of Veterinary Science, University of Queensland. Leukocytes were isolated using the ACCUSPIN System-Histopaque-1077 (Sigma-Aldrich, Australia) according to the manufacturer’s instructions.

### Co-culture of cAT-MSCs and ciMSCs with mitogen-stimulated mixed canine leukocytes

Leukocytes were maintained in medium consisting of RPMI-1640 medium (Sigma-Aldrich) supplemented with 10% fetal calf serum (FCS; JRH Biosciences, Australia), 2 mM L-glutamine, 0.1 mM NEAA, 1 mM sodium pyruvate (Gibco), 10,000 units/ml penicillin and 10,000 µg/ml streptomycin (1% Pen-Strep) (Gibco), and 50 µM 2-mercaptoethanol (Gibco), in flat-bottom 24-well cell culture plates (Costar). The following co-cultures were established, in independent duplicate samples, in 1 ml of the medium described above in a 24-well cell culture plate: (a) 1 × 10^6^ leukocytes and 1 × 10^5^ cAT-MSCs; (b) 1 × 10^6^ leukocytes and 1 × 10^5^ ciMSCs; (c) 1 × 10^6^ leukocytes; (d) 1 × 10^5^ cAT-MSCs and (e) 1 × 10^5^ ciMSCs. Concanavalin A (Sigma-Aldrich), a mitogenic stimulant, was added at a concentration of 25 µg/ml to stimulate the proliferation of the T lymphocytes. Cultures were maintained at 37 °C in 5% CO_2_ for 72 h.

After 72 h, leukocytes were precipitated from the cell culture medium by centrifugation at 200 × g for 2 min before freezing at − 80 °C. The culture medium from all wells was frozen at -80 °C for future analysis. cAT-MSCs and ciMSCs were enzymatically collected with TrypLE Express (Gibco) and the cell pellets stored at − 80 °C.

### Enzyme-linked immunoassays

Culture supernatants were used to determine the concentration of TGF-β1, VEGF, IL-8 and IL-1β in the different co-culture groups. Canine-specific Quantikine ELISA kits for TGF-β1 (R&D Systems, USA), IL-8 (R&D Systems), VEGF (R&D Systems) and IL-1β (R&D Systems) were used according to the manufacturer’s instructions. Each sample was assayed in triplicate. Plates were analysed with an Infinite M200 (Tecan, Switzerland) microplate reader at the Australian National Fabrication Facility (ANFF, The University of Queensland, Brisbane, Australia).

### Statistical analysis

Results are presented as the mean ± standard error of the mean (SEM). The comparative analysis between treatment groups was conducted using one-way ANOVA and the means were compared with Student’s t-test using the GraphPad7 Prism software (San Diego, CA, USA). Significance is defined as: ns = not significant *p* > 0.05; *p ≤ 0.05; **p ≤ 0.005; ***p ≤ 0.0002; ****p ≤ 0.0001.

### Ethics approval and consent to participate

The collection and use of animal tissues was approved by the Animal Ethics Committee at The University of Queensland under ethics approval numbers SVS/194/15, SVS/099/17 and SVS/ANRFA/453/18.

## Results

### Sequencing of ciMSC, cBM-MSC and ciPSC transcriptomes

**Figure 1:**
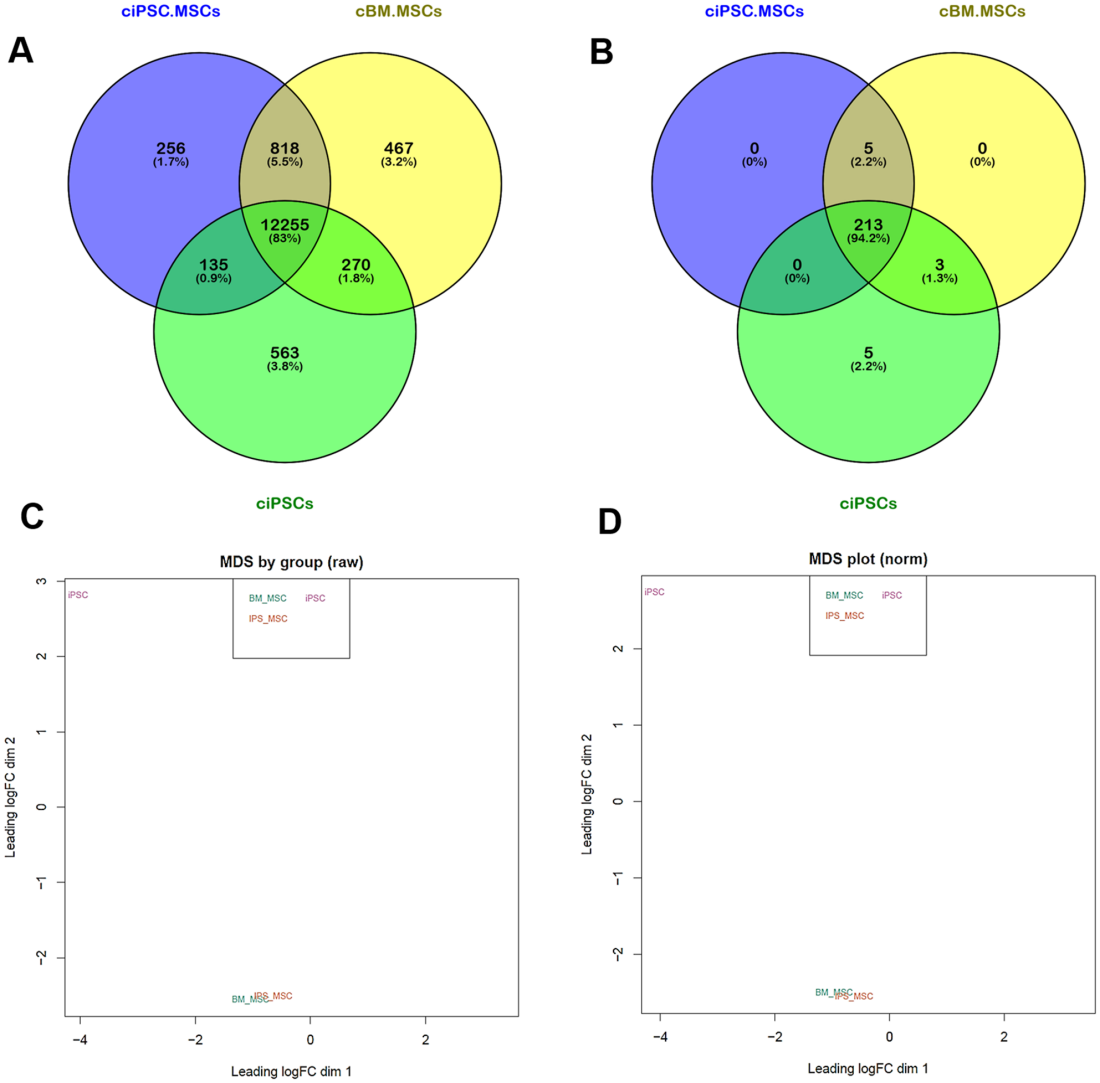

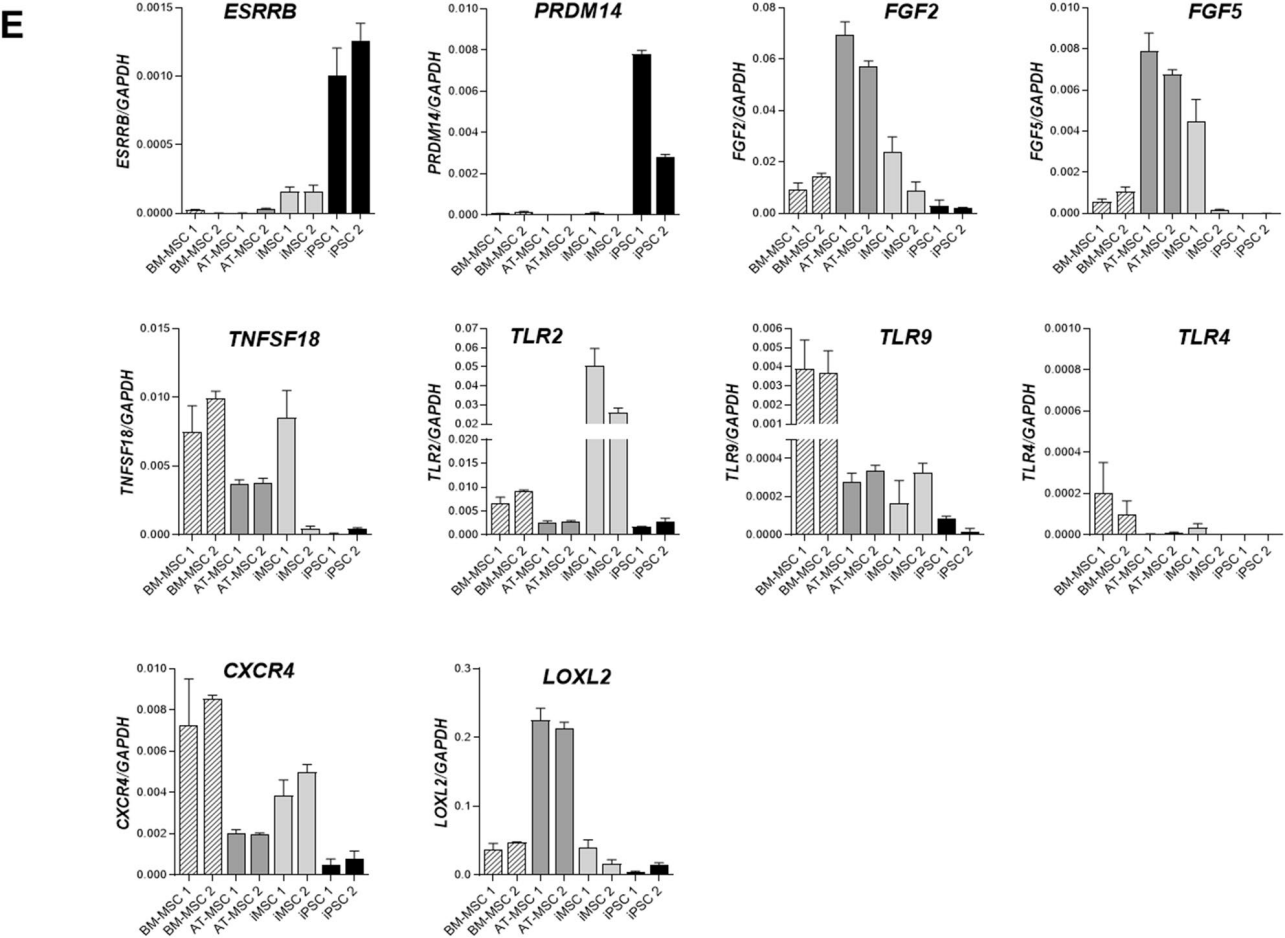
Canine iPSC-derived MSCs more closely resemble harvested bone marrow-derived MSCs than the iPSCs from which they were generated. (**A**) Venn analysis of expressed genes (CPM ≥ 1) identified 83% of the 14,765 genes analysed as being co-expressed by canine bone marrow-derived mesenchymal stem cells (cBM-MSCs), canine induced pluripotent stem cell-derived mesenchymal stem cells (ciPSC-MSCs) and canine induced pluripotent stem cells (ciPSCs). 135 genes, representing 0.9%, are shared by ciPSC-MSCs and the ciPSCs, while the ciPSC-MSCs and cBM-MSCs share 818 (5.5%) genes in common. (**B**) Venn analysis using the transcriptome data for 224 pluripotency factors showed that ciPSC-MSCs, cBM-MSCs and ciPSCs co-express 213 (94.2%) of these pluripotency factors. (**C**) Multidimensional scaling (MDS) plots using both raw gene count and (**D**) normalised gene count data placed the ciPSC-MSCs and cBM-MSCs as a superimposed cluster while the ciPSCs formed a separate cluster. (**E**) Quantitative RT-PCR analysis of the expression of key genes identified from the RNAseq as being differentially expressed between the cMSCs and ciPSCs. Both the RNAseq and qRT-PCR data point to the ciMSCs as being more similar in their transcriptional profiles to AT- and BM-MSCs than to the ciPSCs from which they were derived.

Venn analysis of expressed genes (CPM ≥ 1) identified 83% of the 14,765 canine genes analysed as being co-expressed by all three cell types, including the cell surface markers *CD73 (NT5E)*, *CD90 (THY-1)* and *CD105 (ENDOG)*, confirming our original data^42^, in addition to *CD-44* (Fig. 1a, Table 1 and Supplementary data 1a). Only 135 genes, representing 0.9%, were shared exclusively by the ciMSCs and the ciPSCs (Fig. 1a and Supplementary data 1a). In contrast, 818 genes (5.5%) were expressed exclusively by the ciMSCs and cBM-MSCs including the cell surface proteins *ANPEP* (*CD-13*) and *PDGFRA* (*CD-140a*), cytokines *FGF-2, FGF-5, IL13RA1, LEPR, NOV, PTN, SLIT-1* and *TNC*, and tumour necrosis factors *TNFSF-13* and *TNFSF-18* (Fig. 1a, Table 1 and Supplementary data 1a). cBM-MSCs also expressed the cytokines *EDN-2*, *EDN-3*, *SEMA-3A* and *GREM-2,* while ciMSCs expressed *FGF-10* (Supplementary data 1a). Both ciMSCs and cBM-MSCs express the Toll-like receptors *TLR-2* and *TLR-9* (Table 1 and Supplementary data 1a). Expression of the chemokine receptor *CXCR-4,* which is essential for the homing of MSCs to sites of inflammation^43,44^, is also detectable in ciMSCs and cBM-MSCs, but not ciPSCs (Table 1 and Supplementary data 1a). *LOXL-2*, which is involved in epithelial to mesenchymal transition (EMT), is expressed in the ciMSCs, cBM-MSCs and ciPSCs, but at higher levels in the ciMSCs and cBM-MSCs (Supplementary data 1a).

**Table 1:** Key genes expressed by each of ciMSCs, cBM-MSCs and ciPSCs.

Genes expressed by ciMSCs, cBM-MSCs & ciPSCs	Genes expressed by ciMSCs & cBM-MSCs	Genes expressed by ciPSCs
*CD73 (NT5E)* *CD90 (THY-1)* *CD105 (ENDOG)* *CD-44* *DNMT-3A* *DNMT-3B* *JARID-2* *KLF-4* *LEF-1* *LIF* *LIFR* *LIN-28A* *c-MYC* *MYC-L* *NANOG* *NR6A1* *POU2F1* *POU5F1 (OCT-4)* *SF1* *SOX-2* *STAT-3* *TERT* *TFAP2C* *WNT-3* *WNT-5A*	*ACVR1C* *ANPEP* (*CD-13*) *CXCR-4* *FGF-2* *FGF-5* *IL13RA1* *LEPR* *NOV* *PDGFRA* (*CD-140a*) *PTN* *SATB-1* *SLIT-1* *TLR-2* *TLR-9* *TNC* *TNFSF-13* *TNFSF-18*	*CCND-1* *ESRRB* *FGF-4* *PRDM-14* *TLE-2*

Venn analysis using the transcriptome data for 224 pluripotency factors demonstrated that ciMSCs, cBM-MSCs and ciPSCs co-express 213 (94.2%) of these factors including *DNMT-3A, DNMT-3B, JARID-2, KLF-4, LEF-1, LIF, LIFR, LIN-28A, c-MYC, MYC-L, NANOG, NR6A1, POU2F1, POU5F1 (OCT-4), SF1, SOX-2, STAT-3, TERT, TFAP2C, WNT-3* and *WNT-5A* (Fig. 1b, Table 1 and Supplementary data 1b)*. ESRRB* and *PRDM-14*, both of which are associated with naïve, rather than primed, pluripotency were expressed only in the ciPSCs (Table 1 and Supplementary data 1b). Also unique to the ciPSCs was the expression of *CCND-1, FGF-4* and *TLE-2* (Table 1 and Supplementary data 1b). Three factors associated with pluripotency were expressed only by the ciMSCs and cBM-MSCs: *ACVR1C, FGF-5* and *SATB-1* (Table 1 and Supplementary data 1b). Multidimensional scaling (MDS) plots, using both raw gene count (Fig. 1c) and normalised gene count (Fig. 1d) data, placed the ciMSCs and cBM-MSCs as a superimposed cluster while the ciPSCs formed a separate cluster.

Due to financial constraints we were only able to perform RNAseq on one sample from each of the ciMSCs, cBM-MSCs, and ciPSCs; thus, we used quantitative RT-PCR to examine the expression of 10 key genes, identified from the RNAseq as being differentially expressed between the cMSCs and ciPSCs, in 2 independent biological samples from each of the ciMSCs, cAT-MSCs, cBM-MSCs and ciPSCs. Our previous work^42^ identified that ciMSCs and cBM-MSCs express the core pluripotency factors *POU5F1/OCT-4*, *SOX-2* and *NANOG*, which was confirmed by the RNAseq analysis in this study, and so we focused the qRT-PCR analysis on the two genes that are specific for naïve pluripotency: *ESRRB* and *PRDM-14*. We also examined the expression of 8 genes that were differentially expressed between the cMSCs and ciPSCs in the RNAseq analysis and which are associated with important pathways in MSCs, specifically: *FGF-2, FGF-5, TNFSF-18, TLR-2, TLR-4, TLR-9, CXCR-4* and *LOXL-2*.

Supporting the RNAseq data, *ESRRB* and *PRDM-14* are expressed at appreciable levels only by the ciPSCs (Fig. 1e). Similarly, *FGF2* expression is significant only within the ciMSCs, cAT-MSCs and cBM-MSCs with barely detectable levels of expression in the ciPSCs (Fig. 1e). Expression of *FGF5* is similarly restricted to the harvested cMSCs and ciMSCs, with no detectable expression in the ciPSCs which is in keeping with the results from the RNAseq; however, the expression level in one of the two lines of iMSCs is also very low (Fig. 1e). *TNFSF-18* is robustly expressed by the cAT-, cBM- and ciMSCs and not by the ciPSCs, but again, the expression level in one of the two lines of iMSCs is very low (Fig. 1e). Both the ciMSCs and cBM-MSCs express *TLR-2* and *TLR-9*, as seen in the RNAseq analysis (Fig. 1e). In contrast, the cAT-MSCs express barely detectable levels of *TLR-2* (Fig. 1e). A surprising finding from the RNAseq data was that neither the ciMSCs nor the cBM-MSCs expressed *TLR-4*. This observation is supported by the qRT-PCR data, which also demonstrates a lack of *TLR-4* expression in the AT-MSCs (Fig. 1e). Significant expression of *CXCR-4* is found in ciMSCs, cAT-MSCs and cBM-MSCs but not in ciPSCs (Fig. 1e). Levels of *LOXL-2* expression are higher in ciMSCs and cBM-MSCs than in ciPSCs, with the highest levels in the cAT-MSCs (Fig. 1e). Thus, both the RNAseq and qRT-PCR data point to the ciMSCs as being more similar in their transcriptional profiles to AT- and BM-MSCs than to ciPSCs.

### ciMSCs constitutively express immunomodulatory and anti-inflammatory factors and respond to priming with pro-inflammatory cytokines

**Figure 2:**
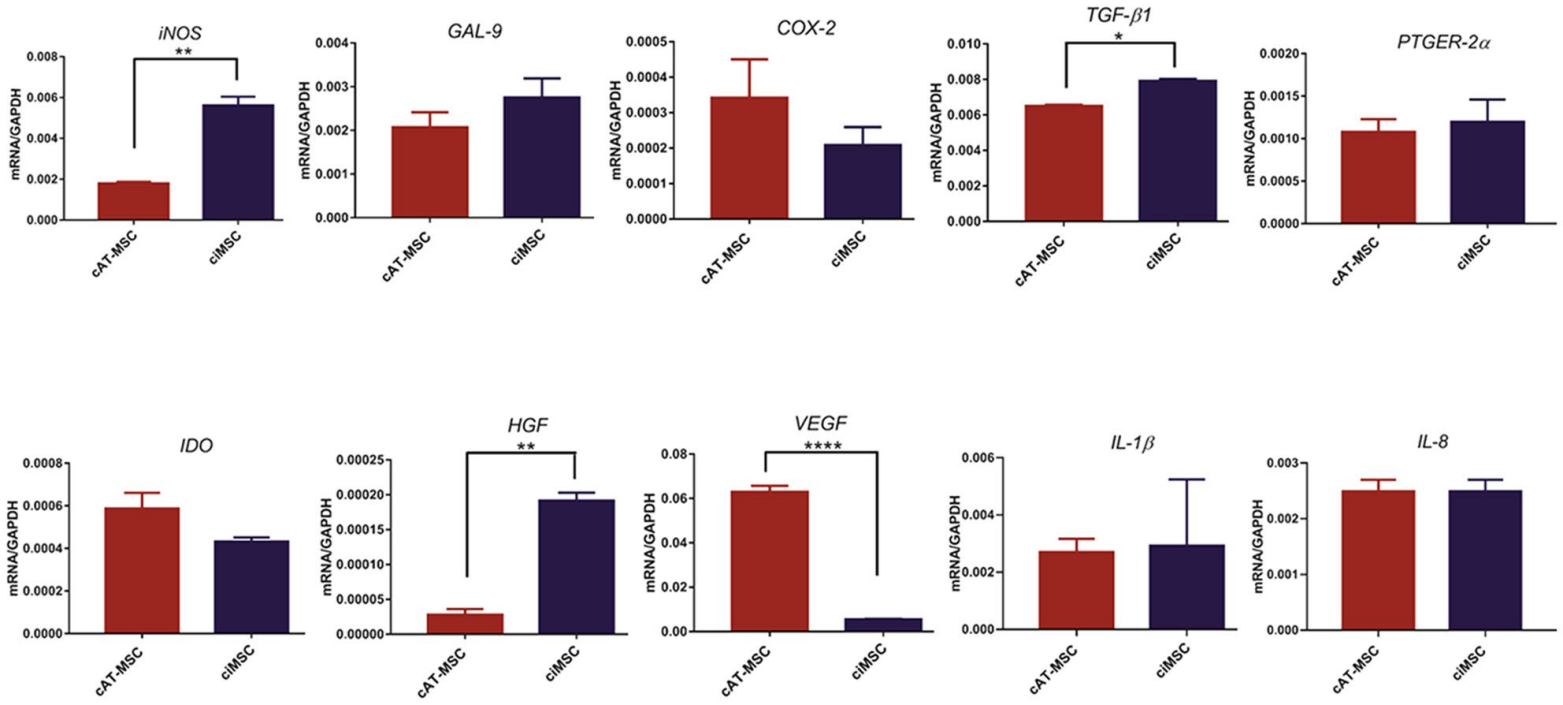
ciMSCs and cAT-MSCs constitutively express immunomodulatory and anti-inflammatory factors. ciMSCs and cAT-MSCs have similar constitutive expression profiles, although ciMSCs express higher levels of *iNOS* and *HGF*, and lower levels of *VEGF,* than the cAT-MSCs. Inducible nitric oxide (*iNOS*); Indoleamine 2,3 dioxygenase (*IDO*); Galectin-9 (*GAL-9*); Cyclooxygenase-2 (*COX-2*); Transforming growth factor-β1 (*TGF-β1*); Prostaglandin receptor-2α (*PTGER-2α*); Hepatocyte growth factor (*HGF*); Vascular endothelial growth factor (*VEGF*) ; Interleukin-8 (*IL-8*) and Interleukin-1β (*IL-1β*). Significance is defined as: ns = not significant *p* > 0.05; *p ≤ 0.05; **p ≤ 0.005; ***p ≤ 0.0002; ****p ≤ 0.0001.

ciMSCs constitutively expressed the immunomodulatory factors *inducible nitric oxide synthase (iNOS), galectin-9 (GAL-9), transforming growth factor-β1 (TGF-β1), prostaglandin receptor-2α (PTGER-2α)* and *vascular endothelial growth factor (VEGF)*, and the pro-inflammatory factors *cyclooxygenase-2 (COX-2), interleukin-1β (IL-1β)* and *interleukin-8 (IL-8)* (Fig. 2)*.* cAT-MSCs had a similar constitutive expression profile, although they expressed *iNOS* and *HGF* at significantly lower levels (Supplementary Table 2), and *VEGF* at significantly higher levels (Supplementary Table 2), than ciMSCs (Fig. 2).

**Figure 3:**
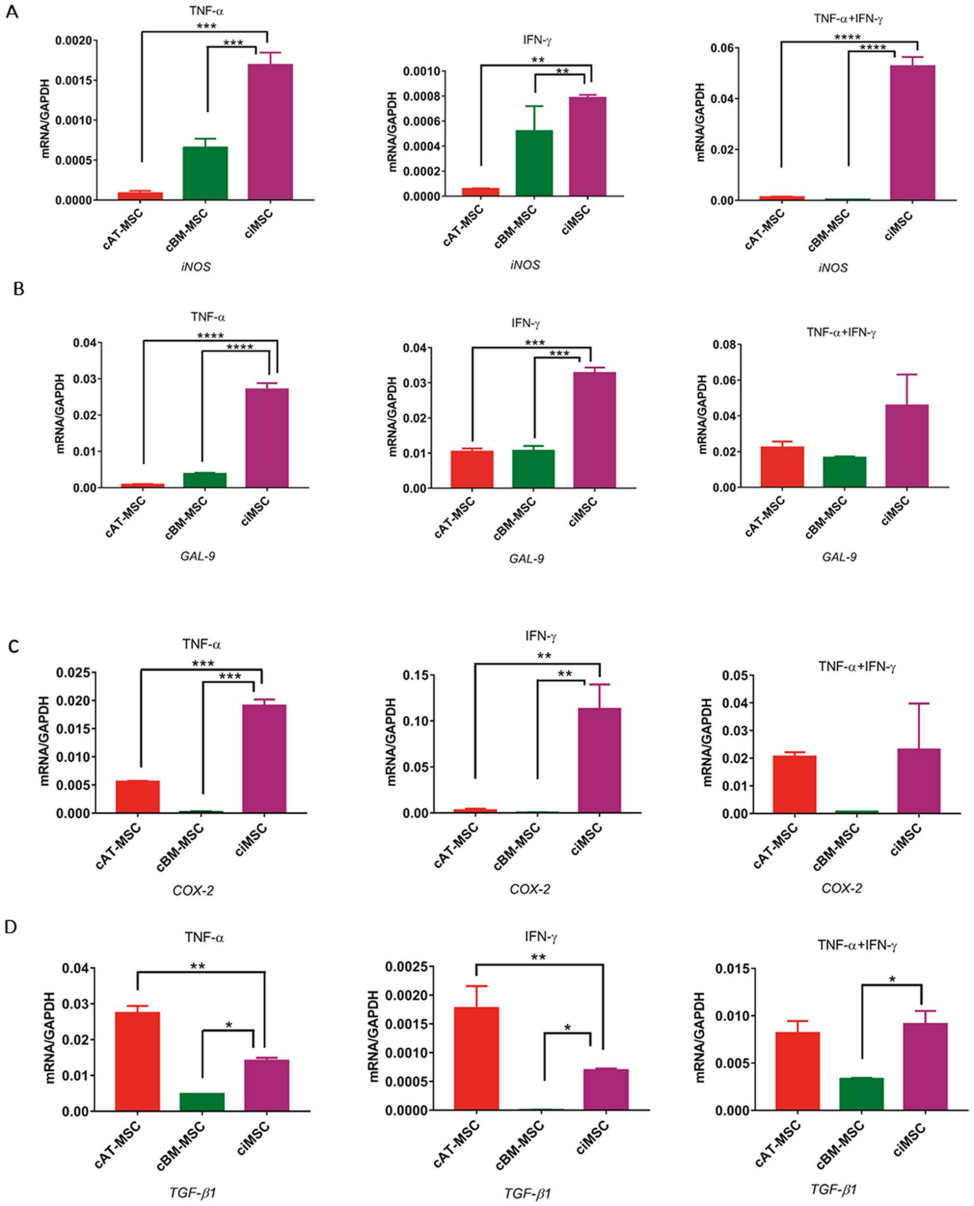

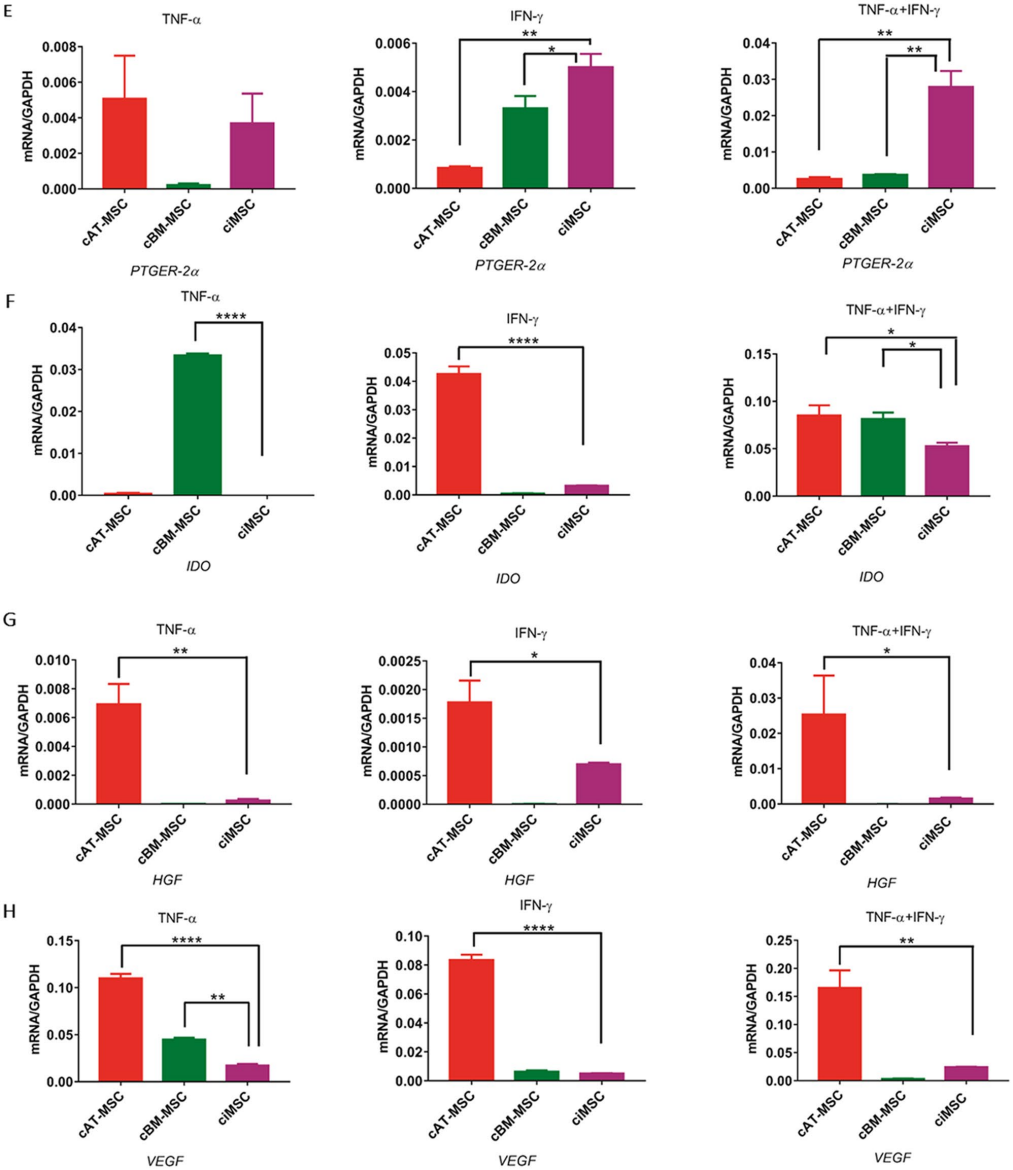

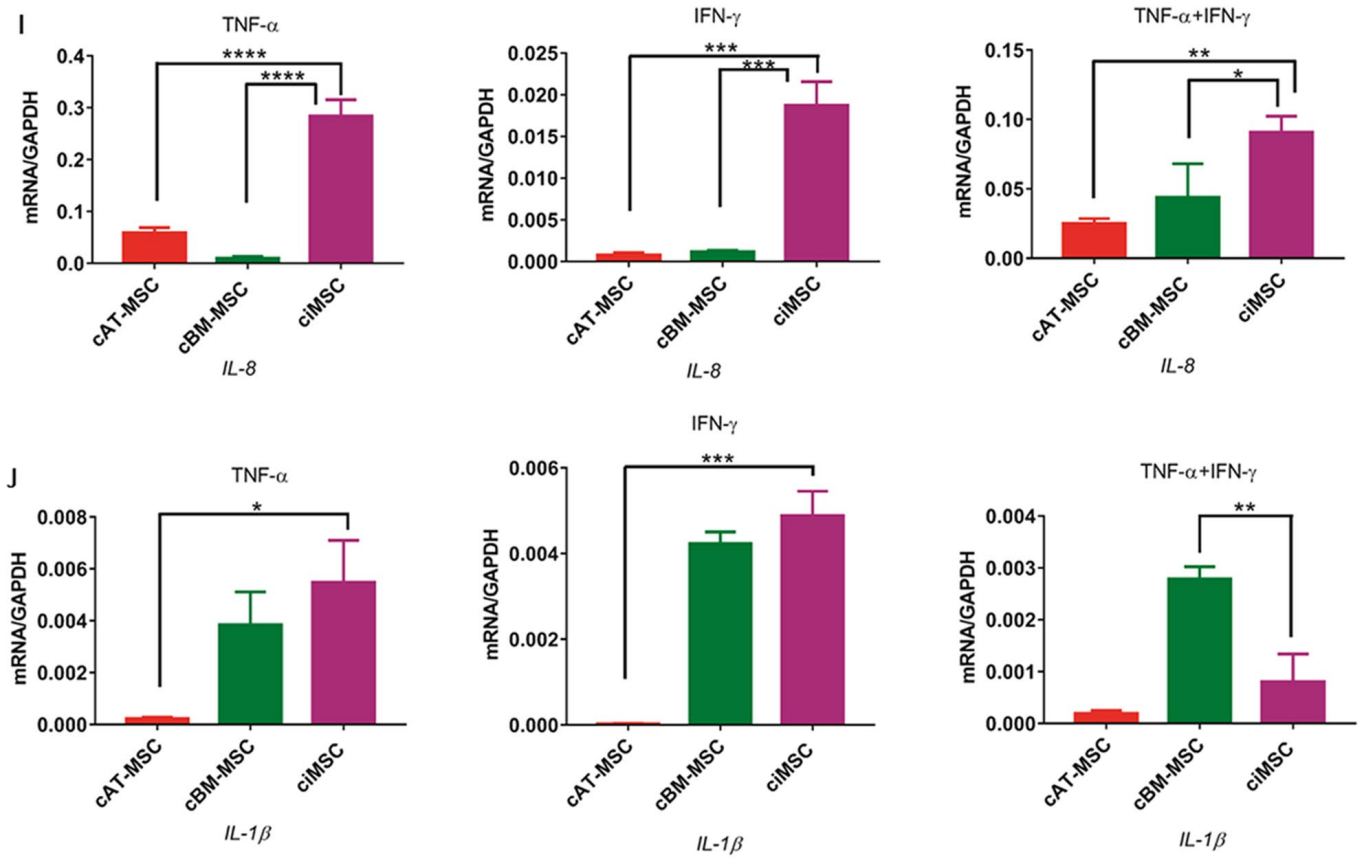
Response of ciMSCs, cAT-MSCs and cBM-MSCs to priming with pro-inflammatory cytokines canine tumor necrosis factor-α (cTNF-α), canine interferon-γ (cIFN-γ) and a combination of both (cTNF-α + cIFN-γ). When stimulated with cTNF-α, cIFN-γ, or a combination of both, ciMSCs upregulated their expression of : (**A**) *iNOS*; (**B**) *GAL-9;* (**C**) *COX-2;* (**D**) *TGF-β;* (**E**) *PTGER-2α;* (**F**) *IDO;* (**G**) *HGF;* (**H**) *VEGF;* (**I**) *IL-8* and (**J**) *IL-1β.* Significance is defined as: ns = not significant *p* > 0.05; *p ≤ 0.05; **p ≤ 0.005; ***p ≤ 0.0002; ****p ≤ 0.0001.

Expression of *iNOS* by ciMSCs was significantly higher than for cAT-MSCs and cBM-MSCs in all three treatment groups with cTNF-α, cIFN-γ or a combination of both (cTNF-α/cIFN-γ) (Fig. 3a; Supplementary Table 2). While expression of *iNOS* by ciMSCs and cAT-MSCs decreased significantly from constitutive levels when treated with cTNF-α and cIFN-γ (Fig. 3a; Supplementary Table 2), when cultured with both in combination (cTNF-α/cIFN-γ) the expression of *iNOS* increased almost tenfold in ciMSCs but remained unchanged in cAT-MSCs (Fig. 3a; Supplementary Table 2).

ciMSCs expressed higher levels of *GAL-9* than cAT-MSCs and cBM-MSCs in response to cTNF-α and cIFN-γ (Fig. 3b; Supplementary Table 2), and upregulated their expression by around 10–20 fold as compared to constitutive levels across all three treatment groups (Fig. 3b; Supplementary Table 2). Expression of *COX-2* was similarly higher in ciMSCs as compared to cAT-MSCs and cBM-MSCs when treated with cTNF-α and cIFN-γ (Fig. 3c; Supplementary Table 2). While cBM-MSCs expressed *COX-2* at barely detectable levels across all three treatment groups, both cAT-MSCs and ciMSCs upregulated their expression by approximately 100 fold in response to cTNF-α/cIFN-γ (Fig. 3c; Supplementary Table 2).

cAT-MSCs and ciMSCs expressed similar levels of *TGF-β1* constitutively (Fig. 2), but cAT-MSCs showed a stronger transcriptional response to cTNF-α and cIFN-γ than did ciMSCs, while they expressed similar levels of upregulated transcription when exposed to combined cTNF-α/cIFN-γ (Fig. 3d; Supplementary Table 2). Expression of *PTGER-2α* was highest in ciMSCs as compared to cAT-MSCs and cBM-MSCs, and was most significantly upregulated when they were cultured with cIFN-γ and cTNF-α/cIFN-γ (Fig. 3e; Supplementary Table 2). Only cBM-MSCs showed detectable expression of *indoleamine 2, 3 dioxygenase (IDO)* in cultures with cTNF-α, while cAT-MSCs expressed the highest levels when cells were exposed to cIFN-γ; however, all three types of MSCs responded to stimulation with cTNF-α/cIFN-γ (Fig. 3f; Supplementary Table 2).

Expression of *HGF* was restricted predominantly to cAT-MSCs across all three treatment groups (Fig. 3g; Supplementary Table 2). Similarly, *VEGF* was also most strongly expressed by cAT-MSCs, with significantly lower levels of expression detected in cBM-MSCs and ciMSCs (Fig. 3h; Supplementary Table 2). While cAT-MSCs and ciMSCs expressed similar levels of *IL-8* constitutively, the ciMSCs showed the most increased response to all three treatments, with the strongest response to cTNF-α (Fig. 3i; Supplementary Table 2). Expression of IL*-1β* remained relatively unchanged in ciMSCs cultured with cTNF-α, cIFN-γ and cTNF-α/cIFN-γ; similarly, cBM-MSCs maintained consistent levels of expression across all three treatment groups (Fig. 3j; Supplementary Table 2). In contrast, cAT-MSCs significantly downregulated their expression compared to constitutive levels (Fig. 3j; Supplementary Table 2).

### Effect of mitogen-stimulated canine lymphocytes on inflammatory cytokine expression of MSCs

**Figure 4:**
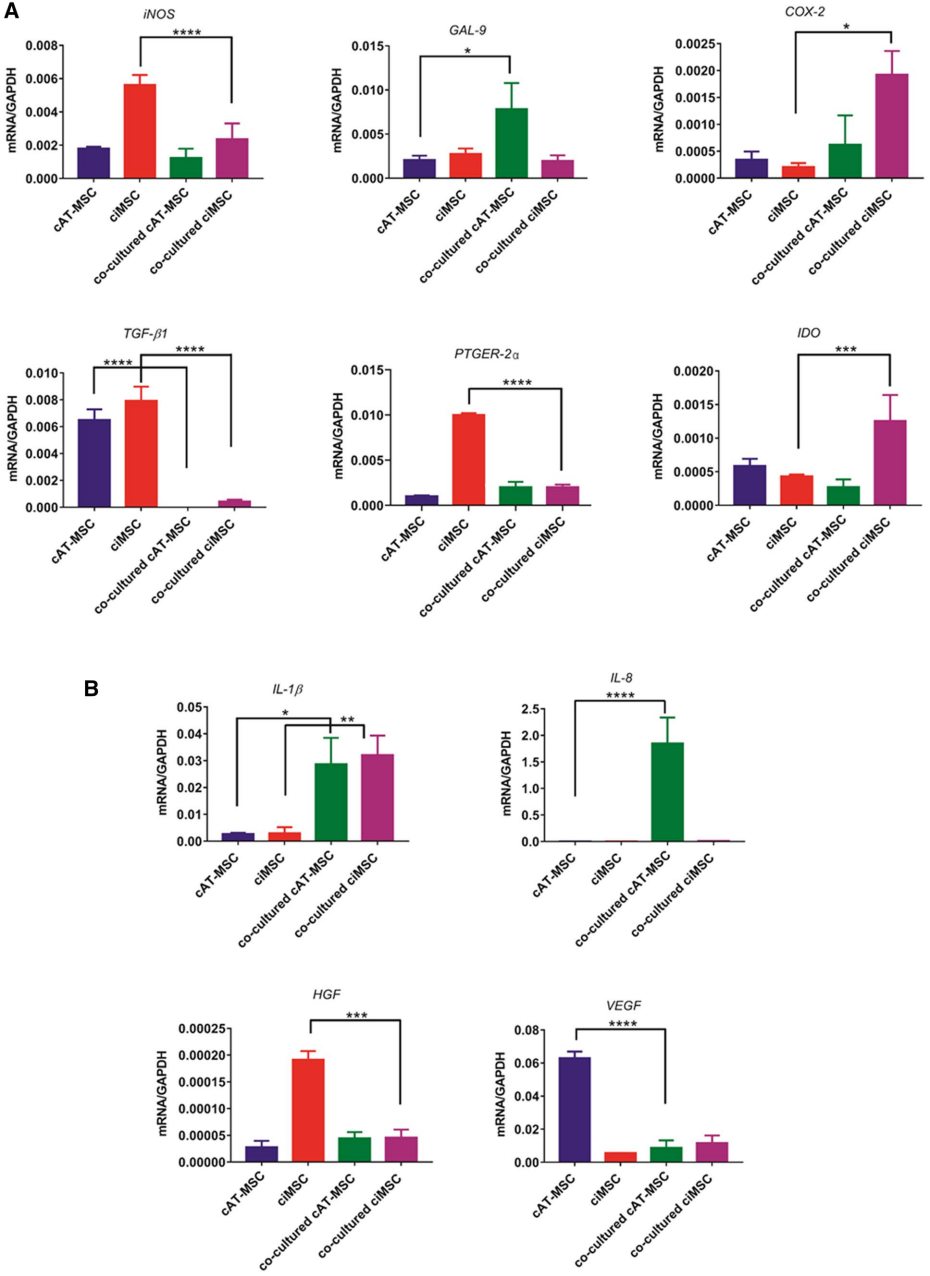
Effect of mitogen-stimulated canine lymphocytes on inflammatory cytokine expression of ciMSCs and cAT-MSCs. When co-cultured with mitogen-stimulated lymphocytes, ciMSCs downregulated their expression of *iNOS*, *HGF*, *TGF-β1*and *PTGER-2α,* while increasing their expression of *COX-2, IDO* and *IL-1β*. Significance is defined as: ns = not significant *p* > 0.05; *p ≤ 0.05; **p ≤ 0.005; ***p ≤ 0.0002; ****p ≤ 0.0001.

When co-cultured with mitogen-stimulated lymphocytes, ciMSCs significantly downregulated their expression of *iNOS, TGF-β1, HGF* and *PTGER-2α* (Fig. 4; Supplementary Table 3). Although *HGF* expression levels also significantly decreased, transcription levels in the control cultures were so low that they are likely not indicative of expression (Fig. 4; Supplementary Table 3)*.* cAT-MSCs downregulated their expression of *TGF-β1* and *VEGF*, while *iNOS* and *PTGER-2α* remained unchanged (Fig. 4; Supplementary Table 3). In response to co-culture, ciMSCs upregulated their expression of *COX-2* and *IDO*, and both ciMSCs and cAT-MSCs increased their expression of *IL-1β* (Fig. 4; Supplementary Table 3). Expression of *GAL-9* and *IL-8* increased in cAT-MSCs but remained unchanged in ciMSCs, while expression of *VEGF* decreased in cAT-MSCs and was unchanged in ciMSCs (Fig. 4; Supplementary Table 3).

### Effect of MSCs on inflammatory cytokine expression of mitogen-stimulated canine lymphocytes

**Figure 5:**
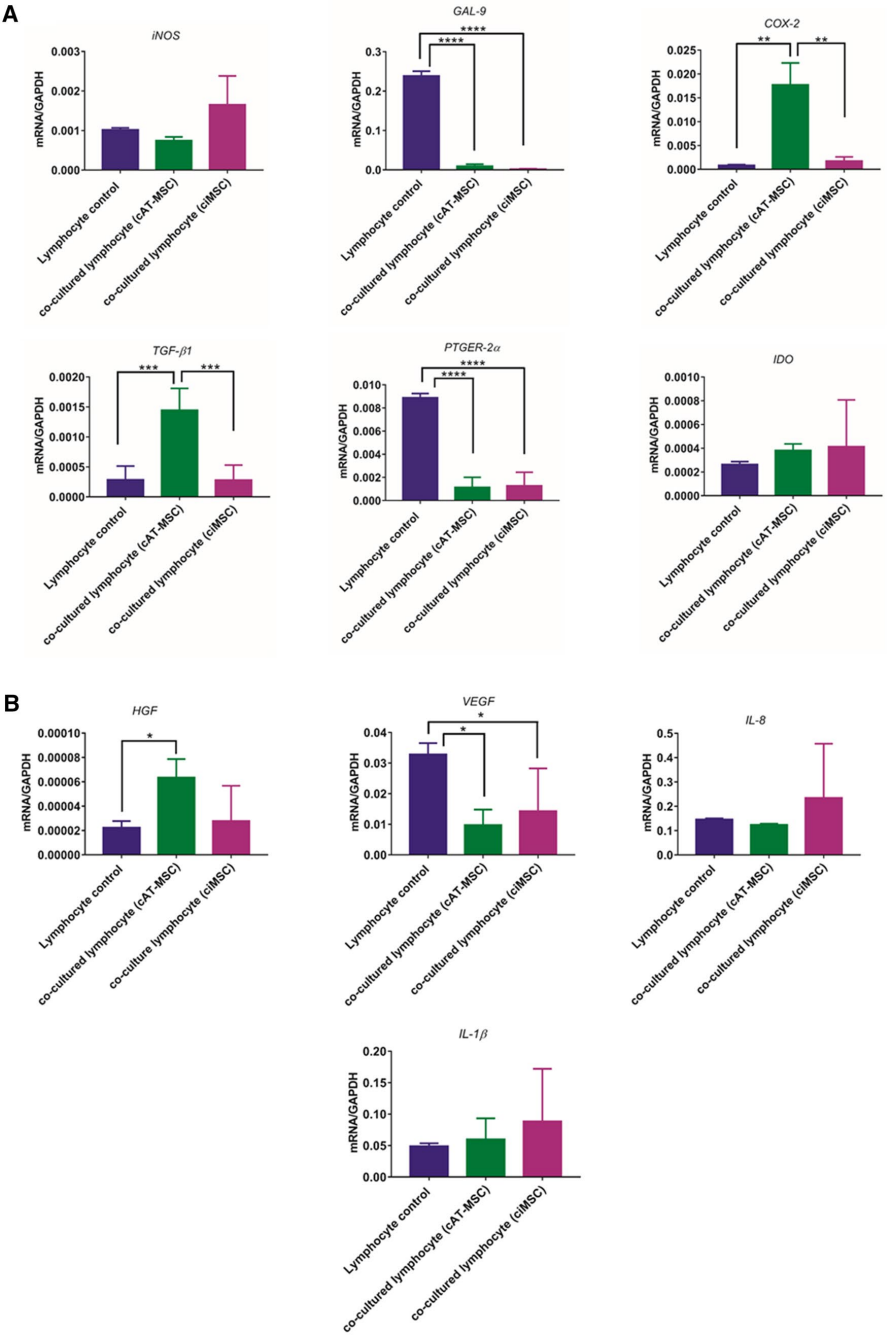
Effect of ciMSCs and cAT-MSCs on inflammatory cytokine expression of mitogen-stimulated canine lymphocytes. Lymphocytes cultured with ciMSCs and cAT-MSCs downregulated their expression of *GAL-9, PTGER-2α* and *VEGF,* while the expression of *iNOS, IDO, IL-8* and *IL-1β* were unchanged. Lymphocytes co-cultured with cAT-MSCs increased their expression of *COX-2* and *TGF-β1.* Significance is defined as: ns = not significant; *p* > 0.05; *p ≤ 0.05; **p ≤ 0.005; ***p ≤ 0.0002; ****p ≤ 0.0001.

Lymphocytes cultured with ciMSCs and cAT-MSCs downregulated their expression of *GAL-9, PTGER-2α* and *VEGF,* while the expression of *iNOS, IDO, IL-8* and *IL-1β* were unchanged (Fig. 5; Supplementary Table 4). In contrast, lymphocytes co-cultured with cAT-MSCs increased their expression of *COX-2, TGF-β1* and possibly *HGF*, although expression levels are so low as to be near the detection threshold (Fig. 5; Supplementary Table 4).

### Effects of co-culture on the secretion of factors by lymphocytes and MSCs

**Figure 6:**
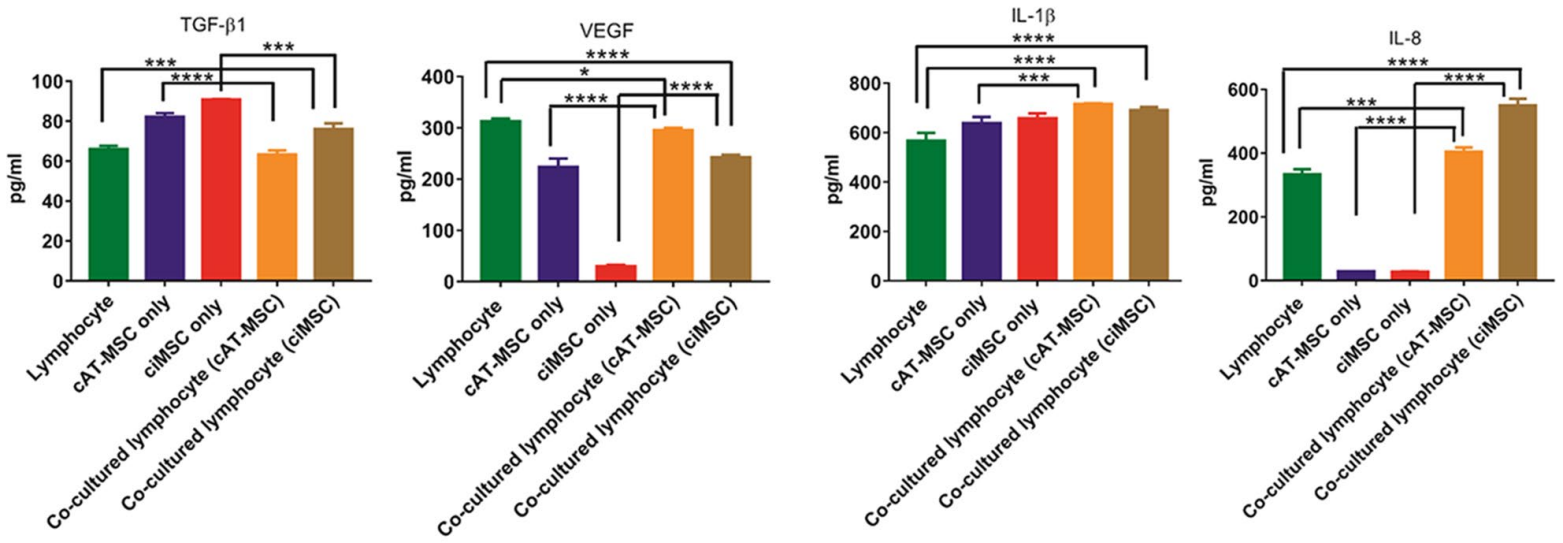
Effects of co-culture on the secretion of factors by lymphocytes, ciMSCs and cAT-MSCs. Lymphocytes, ciMSCs and cAT-MSCs produce IL-1β, IL-8, TGF-β1 and VEGF. Based on mRNA levels (see Figs. 5 and 6), the increase in IL-1β measured in the medium from co-cultures of cMSCs and lymphocytes is likely produced by the ciMSCs and cAT-MSCs rather than the lymphocytes. When similarly referenced to mRNA levels, cAT-MSCs and lymphocytes in co-culture upregulate their expression of IL-8 while ciMSCs do not. Significance is defined as: *p ≤ 0.05; **p ≤ 0.005; ***p ≤ 0.0002; ****p ≤ 0.0001.

The concentrations of canine IL-1β, IL-8, TGF-β1 and VEGF were measured in the supernatant collected from cultures of lymphocytes, ciMSCs and cAT-MSCs, and co-cultures of lymphocytes with each of ciMSCs and cAT-MSCs. In agreement with the qRT-PCR data, both ciMSCs and cAT-MSCs produce IL-1β, IL-8, TGF-β1 and VEGF (Fig. 6; Supplementary Table 5). Furthermore, the relative expression levels of the genes between the two types of MSCs is reflected at the protein level with *VEGF* RNA and protein expression significantly higher in cAT-MSCs as compared to ciMSCs, while all other factors are expressed at similar levels for both RNA and protein (Fig. 6; Supplementary Table 5). Lymphocytes similarly produce all four factors (Fig. 6; Supplementary Table 5).

Based on the qRT-PCR data that showed lymphocytes did not alter their transcription of *IL-1β* in response to co-culture with either ciMSCs or cAT-MSCs, but both types of MSCs increased their transcription of *IL-1β* when co-cultured, the increase in IL-1β measured in the medium from co-cultures is likely produced by the ciMSCs and cAT-MSCs rather than the lymphocytes (Fig. 6; Supplementary Table 5). In contrast, based on the qRT-PCR data, the increase in IL-8 in co-cultures is more likely from the cAT-MSCs and lymphocytes than from the ciMSCs (Fig. 6; Supplementary Table 5).

Both ciMSCs and cAT-MSCs downregulated their expression of *TGF-β1* when co-cultured, while lymphocyte expression, which was lower than that observed in the MSCs, increased or remained unchanged, when co-cultured with cAT-MSCs and ciMSCs, respectively. Thus, lower levels of TGF-β1 were measured in the medium of co-cultured ciMSCs and cAT-MSCs than when the cells were cultured alone, and are similar to the levels detected in medium from lymphocyte cultures (Fig. 6; Supplementary Table 5).

Both cAT-MSCs and lymphocytes expressed significantly higher levels of *VEGF* than ciMSCs and downregulated their expression in co-culture. This dynamic is reflected at the protein level where cAT-MSC/lymphocyte co-cultures have VEGF levels in between the levels for each when cultured separately, and the measurement for ciMSC/lymphocyte co-cultures are higher than the ciMSCs cultured alone but lower than the levels measured for lymphocytes or cAT-MSC/lymphocyte co-cultures (Fig. 6; Supplementary Table 5).

## Discussion

In this study we compared the transcriptome of ciMSCs with cAT-MSCs, cBM-MSCs and ciPSCs and show expression of key pluripotency factors by all cell types*.* Previous studies have similarly demonstrated the expression of pluripotency factors by canine MSCs isolated from adipose tissue^45^, bone marrow^42,45^ and amniotic fluid^46^. In contrast, *ESRRB* and *PRDM-14*, bother than primed, pluripotency^47–50^ are expressed only in the ciPSCs and not the ciMSCs, cAT-MSCs or cBM-MSCs, which is not surprising since the ciPSCs are pluripotent^42,51^ while all three types of MSCs are multipotent^42^. Also unique to the ciPSCs is the expression of *FGF-4* which, in the mouse embryo, is secreted by the epiblast cells of the inner cell mass (ICM) under transcriptional regulation by Oct-4 and Sox-2^52^ where it is thought to play a role in the development of the embryo through the conversion of the ICM into primitive endoderm^53,54^.

Endogenous and exogenously administered MSCs migrate towards tumours and sites of ischaemia and inflammation in response to a range of signalling molecules including the chemokine stromal cell-derived factor-1 (SDF-1), through interaction with its cognate receptor CXC chemokine receptor 4 (CXCR-4), which is expressed on the surface of MSCs^55–58^. Importantly, when considering future therapeutic applications, our ciMSCs express *CXCR-4*, as do the cAT-MSCs and cBM-MSCs, while it is not expressed by the ciPSCs.

Studies in human MSCs from bone marrow, adipose tissue and umbilical cord blood have demonstrated an important role of signalling through Toll-like receptors (TLRs) in regulating the immunomodulatory effects, migration, proliferation and differentiation of MSCs^59–67^. Typically, human MSCs express high levels of TLR-3 and TLR-4, low levels of TLR-1, TLR-2, TLR-5, TLR-6 and TLR-9, and lack expression of TLR-7, TLR-8 and TLR-10. The expression profile of TLRs in our ciMSCs is very similar to that of the cBM-MSCs and, in a more limited analysis to the AT-MSCs, and reflects the expression profile described in human MSCs with expression of *TLR-1, TLR-2, TLR-3, TLR-5, TLR-6* and *TLR-9*, and no expression of *TLR-7, TLR-8* and *TLR-10*. However, unlike human MSCs, neither the ciMSCs nor the cAT-MSCs or cBM-MSCs expressed *TLR-4*. This lack of *TLR-4* expression is very surprising since TLR-4 signalling is responsible for priming human MSCs towards a pro-inflammatory phenotype, while TLR-3 priming induces an anti-inflammatory response^59,64^. Based on limited studies of various canine cell types (not including MSCs) the expression of *TLR-4* in the dog appears to follow a similar profile to that described for other species^68^ and so we could reasonably expect canine MSCs to similarly express high levels of *TLR-4*. A search of the literature did not yield any insight as to a possible explanation for the lack of *TLR-4* expression in our canine MSCs, except to note that the expression of *TLR-4* by human Wharton’s jelly-derived MSCs appears to be variable^59,69^ and so the lack of *TLR-4* expression in our canine MSCs may reflect a species difference or perhaps an effect of culture conditions.

The transcriptome of our ciMSCs is more similar to that of the cBM-MSCs and cAT-MSCs than that of the ciPSCs. This is in contrast to the data of Chow et al.^70^ whose ciPSC-derived MSCs showed a gene expression profile that was markedly different from that of cAT-MSCs and cBM-MSCs, and much more closely resembled that of the ciPSCs from which they were generated. It is possible that the ciPSCs generated by Chow and colleagues^70^ were in a more primed, rather than naïve, state of pluripotency and that this has affected the nature of the resultant ciPSC-derived MSCs. It is perhaps significant that the ciPSCs that we used to generate our ciMSCs show many of the hallmarks of naïve pluripotency including expression of *ESRRB* and *PRDM-14*.

MSC secretion of either IDO or iNOS, depending on the species, has been shown to suppress T cell proliferation^31,71–74^. In human, IDO is the key mediator of T cell suppression^31,75–79^ while in mouse^78^ and horse^80^ iNOS is the major inhibitor of T cell activation. However, recent reports suggest that *IDO,* in addition to *iNOS,* may be involved in the immunomodulatory roles of equine MSCs^35,37,38^. In this study, both ciMSCs and cAT-MSCs constitutively express *iNOS* and when co-stimulated with cTNF-α and cIFN-γ, ciMSCs upregulated their expression of *iNOS* by tenfold. That cAT-MSCs did not show an increase in *iNOS* expression beyond constitutive levels, and cBM-MSCs expressed very low levels in response to cTNF-α/cIFN-γ, is in keeping with the observations by Chow et al.^70^ that cAT-MSCs and cBM-MSCs do not employ the *iNOS*/NO-mediated pathway for immunosuppression. In contrast, the strong upregulation of *iNOS* expression in ciMSCs is similar to observations in the horse where priming of equine bone marrow-derived MSCs with IFN-γ or TNF-α/IFN-γ similarly induced an upregulation of *iNOS*^81^. Expression of *iNOS* significantly decreased in ciMSCs co-cultured with mitogen-stimulated lymphocytes. This would appear to be at odds with our observation of an upregulation of *iNOS* in ciMSCs exposed to cIFN-γ/cTNF-α. However, previous studies have demonstrated that the production of TNF-α by canine lymphocytes is reduced upon co-culture with cAT-MSCs^34^, and the secretion of IFN-γ by canine lymphocytes is similarly suppressed when co-cultured with cAT-MSCs and cBM-MSCs^82^. Thus, the decrease in *iNOS* expression by ciMSCs co-cultured with lymphocytes may be due to low levels of TNF-α and IFN-γ being produced by the canine lymphocytes, possibly as a consequence of suppression by the ciMSCs.

All three types of MSCs responded to stimulation with cTNF-α/cIFN-γ by upregulating their expression of *IDO*. Kang et al.^34^ similarly observed increased expression of *IDO* in canine AT.MSCs co-cultured with concanavalin-stimulated lymphocytes shown to be secreting cTNF-α and cIFN-γ. In our study, while ciMSCs significantly upregulated their expression of *IDO* when co-cultured with concanavalin-stimulated lymphocytes, the transcript levels of *IDO* decreased in co-cultured cAT-MSCs. This discrepancy between our cAT-MSC data and that of Kang et al.^34^ might reflect insufficient levels of IFN-γ and TNF-α being produced by the lymphocytes to stimulate the AT.MSCs, as discussed in the preceding paragraph.

Following TLR-3 priming, the release of TGF-β1 by activated anti-inflammatory MSCs suppresses the proliferation and secretion of cytokines by T lymphocytes and natural killer cells and also inhibits the stimulatory effect of dendritic cells on T lymphocytes^22,25,82–88^. Constitutive expression of *TGF-β1* by ciMSCs, cAT-MSCs and cBM-MSCs (RNAseq data) is in keeping with the data of other studies^34,82,89^ that have similarly demonstrated the constitutive transcription of *TGF-β1* in cBM-MSCs, cAT-MSCs and ciMSCs, respectively. While cAT-MSCs showed a stronger transcriptional response to cTNF-α and cIFN-γ than ciMSCs, both types of MSCs expressed similar levels of *TGF-β1* mRNA when cultured with combined cTNF-α/cIFN-γ.

IL-8 is an MSC-derived chemokine released at the site of injury to enhance the migration and activation of neutrophils^90,91^. In this study, cAT-MSCs and ciMSCs expressed similar levels of *IL-8* constitutively. The constitutive transcription of *IL-8* has previously been described in canine AT.MSCs and human BM.MSCs^92^. ciMSCs showed the strongest response to all three treatments, particularly to cTNF-α. The induced upregulation of *IL-8* by inflammatory stimuli has also been reported in human^93^ and equine MSCs^81,94^.

## Conclusion

In both their transcriptome and in their functional responses to inflammatory cytokines and mitogen-stimulated lymphocytes, our ciMSCs are highly similar to harvested MSCs, supporting further investigation into their potential therapeutic applications for immune-mediated and inflammatory conditions in the dog.

## Supplementary Information

Supplementary Data.

Supplementary Table 1.

Supplementary Table 2.

Supplementary Table 3.

Supplementary Table 4.

Supplementary Table 5.

## Data Availability

The datasets used and analysed during the current study are available from the corresponding author on reasonable request.

## Abbreviations

ACVR1C: Activin A receptor type 1C
ANPEP: Alanyl aminopeptidase
cAT-MSCs: Canine adipose tissue-derived mesenchymal stromal cells
cBM-MSCs: Canine bone marrow-derived mesenchymal stromal cells
CCN3: Cellular communication network factor 3
CCND-1: Cyclin D-1
CDH-1: Cadherin-1
cIFN-γ: Canine interferon-γ
ciMSCs: Canine induced pluripotent stem cell-derived mesenchymal stromal cells
ciPSCs: Canine induced pluripotent stem cells
cMSCs: Canine mesenchymal stromal cells
COX-2: Cyclooxygenase-2
cTNF-α: Canine tumor necrosis factor-α
CXC-4: Chemokine receptor-4
DNMT-3A: DNA methyltransferase 3 alpha
DNMT-3B: DNA methyltransferase 3 beta
EDN-2: Endothelin-2
EDN-3: Endothelin-3
EMT: Epithelial‐mesenchymal transition
ESRRB: Estrogen related receptor beta
FGF-2: Fibroblast growth factor-2
FGF-4: Fibroblast growth factor-4
FGF-5: Fibroblast growth factor-5
FGF-10: Fibroblast growth factor-10
GADD: Growth arrest and DNA damage
GAL-9: Galectin-9
GREM-2: Gremlin-2
HCK: Hemopoietic cell kinase
HGF: Hepatocyte growth factor
HO1: Haem oxygenase-1
ICM: Inner cell mass
IDO: Indoleamine 2, 3-dioxygenase
IFN-γ: Interferon-γ
IL-1β: Interleukin-1β
IL-8: Interleukin-8
IL13RA: Interleukin 13 receptor subunit alpha
iNOS: Inducible nitric oxide synthase
IRS-1: Insulin receptor substrate 1
JARID-2: Jumonji and AT-rich interaction domain containing 2
KLF-4: Kruppel like factor-4
LEF-1: Lymphoid enhancer binding factor 1
LEPR: Leptin receptor
LIF: Leukemia inhibitory factor
LIFR: Leukemia inhibitory factor receptor
LIN-28A: Lin-28 homolog A
LOXL-2: Lysyl oxidase like 2
MEF-2C: Myocyte enhancer factor-2C
MITF: Melanocyte inducing transcription factor
NEAA: Non-essential amino acid
NR6A1: Nuclear receptor subfamily 6 group A member 1
OCLN: Occludin
OCT-4: Octamer-binding transcription factor 4
PDGFC: Platelet derived growth factor C
PDGFRA: Platelet derived growth factor receptor alpha
PGE2: Prostaglandin E2
POU5F1: POU domain, class 5, transcription factor 1
PRDM-14: PR domain zinc finger protein 14
PTGER-2α: Prostaglandin receptor-2α
PTN: Pleiotrophin
SALL-3: Spalt like transcription factor 3
SATB-1: SATB homeobox-1
SDF1: Stromal cell‐derived factor 1
SEMA-3A: Semaphorin-3A
SF1: Steroidogenic factor 1
SLIT-1: Slit guidance ligand 1
SOX-2: SRY (sex determining region Y)‐box 2
STAT-3: Signal transducer and activator of transcription-3
TERT: Telomerase reverse transcriptase
TFAP2C: Transcription factor AP-2 gamma
TGF-β1: Transforming growth factor-β1
TLE-2: Transducin like enhancer of split-2
TLR-1: Toll-like receptor 1
TLR-2: Toll-like receptor 2
TLR-3: Toll-like receptor 3
TLR-4: Toll-like receptor 4
TLR-5: Toll-like receptor 5
TLR-6: Toll-like receptor 6
TLR-7: Toll-like receptor 7
TLR-8: Toll-like receptor 8
TLR-9: Toll-like receptor 9
TLR-10: Toll-like receptor 10
TNC: Tenascin C
TNF: Tumour necrosis factor
TNFSF-13: TNF superfamily member-13
TNFSF-18: TNF superfamily member-18
VCAM-1: Vascular cell adhesion molecule-1
VEGF: Vascular endothelial growth factor
WNT-3: Wnt family member-3
WNT-5A: Wnt family member-5A
