# Supplementary material for: Pluripotency and immunomodulatory signatures of canine induced pluripotent stem cell-derived mesenchymal stromal cells are similar to harvested mesenchymal stromal cells
Source: Sci Rep. 2021 Feb 10;11:3486. doi: 10.1038/s41598-021-82856-3 (PMC7875972; doi:10.1038/s41598-021-82856-3)
Supplement: Supplementary file 2 — Supplementary Table 1. [file 41598_2021_82856_MOESM2_ESM.docx]

| Gene | Sequence (5’→3’, forward/reverse) |
| --- | --- |
| cGAPDH | AACATCATCCCTGCTTCCAC / GACCACCTGGTCCTCAGTGT* |
| cTGF-β1 | CTCAGTGCCCACTGTTCCTG / TCCGTGGAGCTGAAGCAGA* |
| ciNOS | CCAAGAACGTGTTCACCCTG / TGCAGGGCTGTCTACTACTC* |
| cGAL-9 | GAACGGGAGCCATTTTCGC / GTTTGAGTGATGGGAGCCGA* |
| cCOX-2 | ACATCCTGACCCACTTCAAG / CAGGTCCTCGCTTATGATCT* |
| cPTGER-2α | ACCATCACCTTCGCCATCTG / AACCCGACAACAGAGGACTG* |
| cIL-8 | TGGCAGCTTTTGTCCTTTCT / GTCCAGGCACACCTCATTTC* |
| cIL-1β | TCTGGTAGATGAGGGCATCC / TATCCGCATCTGTTTTGCAG* |
| cIDO | TGGTTTATGCAGACTTGTCTT / AGACAAGTATATGCGAAGAAC* |
| cHGF | ACTGCCGGAATCCTGATGAC / TCTCTGTTTCGAGAGGGGAAA* |
| cVEGF | AGCTGGAGCACTTCAATCCC / GTACTTGCACCACTCGCTCT* |
| cTLR-2 | ATGATGTCTCCTACCCGCCT / GGGGGTTAAAGCTCAGGTCC |
| cTLR-4 | AATACCCCGAGCCTGATGTG / CAGGCTGCTCCAGTTGAAGA |
| cTLR-9 | AATACCCCGAGCCTGATGTG / CAGGCTGCTCCAGTTGAAGA |
| cLOXL-2 | ACGTACCCCCTGGAGACTAC, CCGCCTATGTGGCAGTTGTA |
| cCXCR-4 | GCTCAGGCGACTATGACTCC, TGCCCACTATGCCAGTCAAG |
| cFGF-2 | CCCAAGAGGCTGTACTGCAAA, CGCCTCTCTCTTCTGCTTGAA |
| cFGF-5 | TGCTGTGTCTCAGGGGATTG, ACTTCGGTGTATTGCGGAGG |
| cTNFSF-18 | TCGGAAAGCCTGTTCACCAT, GACGTTGGACTTTTGCTGGG |
| cESRRB | GGAGGCGTGCTAGAGATGAA, GGACTGGTCACCACTAAGGG |
| cPRDM-14 | TCTGAGGAGGGCTCCAAACT, CGCCTTTACCAGAGCTGTCT |

Pluripotency and immunomodulatory signatures of canine induced pluripotent stem cell-derived mesenchymal stromal cells are similar to harvested mesenchymal stromal cells. Arash Shahsavari, Prasanna Weeratunga, Dmitry A. Ovchinnikov, and Deanne J. Whitworth.

**Supplementary Table 1.** Canine primer sets used in this study. Primers denoted with * are from Kang et al., 2008.
