# Supplementary material for: Pluripotency and immunomodulatory signatures of canine induced pluripotent stem cell-derived mesenchymal stromal cells are similar to harvested mesenchymal stromal cells
Source: Sci Rep. 2021 Feb 10;11:3486. doi: 10.1038/s41598-021-82856-3 (PMC7875972; doi:10.1038/s41598-021-82856-3)
Supplement: Supplementary file 3 — Supplementary Table 2. [file 41598_2021_82856_MOESM3_ESM.docx]

Pluripotency and immunomodulatory signatures of canine induced pluripotent stem cell-derived mesenchymal stromal cells are similar to harvested mesenchymal stromal cells. Arash Shahsavari, Prasanna Weeratunga, Dmitry A. Ovchinnikov, and Deanne J. Whitworth.

**Supplementary Table 2.** **Response of ciMSCs, cAT-MSCs and cBM-MSCs to priming with the pro-inflammatory cytokines canine tumor necrosis factor-α (cTNF-α), canine interferon-γ (cIFN-γ) and a combination of both (cTNF-α+cIFN-γ).** Inducible nitric oxide (iNOS); Indoleamine 2,3 dioxygenase (IDO); Galectin-9 (GAL-9); Cyclooxygenase-2 (COX-2); Transforming growth factor-β1 (TGF-β1); Prostaglandin receptor-2α (PTGER-2α); Hepatocyte growth factor (HGF); Vascular endothelial growth factor (VEGF) ; Interleukin-8 (IL-8) and Interleukin-1β (IL-1β). (a) induced pluripotent stem cell-derived MSCs (ciMSCs) vs. adipose tissue-derived MSCs (cAT-MSCs); (b) ciMSCs vs. bone marrow-derived MSCs (cBM-MSCs); (c) cAT-MSCs vs. cBM-MSCs; (d) ciMSCs vs. cAT-MSCs. Not significant (NS) p> 0.05; * p≤0.05; ** p≤0.005; *** p≤0.0002; **** p≤0.0001.

| Cytokine | ciMSCs | SEM | cAT-MSCs | SEM | cBM-MSCs | SEM | *p value* |
| --- | --- | --- | --- | --- | --- | --- | --- |
| ***iNOS*** |  |  |  |  |  |  |  |
| TNF-α | 0.001 | 0.0001 | 0.00008 | 0.00003 | 0.0006 | 0.0001 | a*** b*** c* |
| IFN-γ | 0.0007 | 0.00002 | 0.00005 | 0.0000001 | 0.0005 | 0.0001 | a** b** c* |
| TNF-α+IFN-γ | 0.05 | 0.003 | 0.001 | 0.0001 | 0.0004 | 0.00003 | a**** b**** c ^NS^ |
| Control | 0.005 | 0.0004 | 0.001 | 0.00007 | NA | NA | d**** |
| ***IDO*** |  |  |  |  |  |  |  |
| TNF-α | 0.0000001 | 0.00000001 | 0.0004 | 0.0002 | 0.03 | 0.0004 | a ^NS^ b**** c**** |
| IFN-γ | 0.003 | 0.0001 | 0.04 | 0.002 | 0.0004 | 0.0001 | a**** b ^NS^ c**** |
| TNF-α+IFN-γ | 0.05 | 0.003 | 0.08 | 0.01 | 0.08 | 0.006 | a* b* c ^NS^ |
| Control | 0.0004 | 0.00001 | 0.0005 | 0.00007 | NA | NA | d ^NS^ |
| ***GAL-9*** |  |  |  |  |  |  |  |
| TNF-α | 0.02 | 0.001 | 0.0008 | 0.0001 | 0.003 | 0.0002 | a**** b**** c ^NS^ |
| IFN-γ | 0.03 | 0.001 | 0.01 | 0.0008 | 0.01 | 0.001 | a*** b*** c ^NS^ |
| TNF-α+IFN-γ | 0.04 | 0.01 | 0.02 | 0.003 | 0.01 | 0.0006 | a ^NS^ b ^NS^ c ^NS^ |
| Control | 0.002 | 0.0004 | 0.002 | 0.0003 | NA | NA | d ^NS^ |
| ***COX-2*** |  |  |  |  |  |  |  |
| TNF-α | 0.019 | 0.001 | 0.005 | 0.0001 | 0.0002 | 0.00009 | a*** b**** c** |
| IFN-γ | 0.1 | 0.02 | 0.002 | 0.001 | 0.0004 | 0.0002 | a** b** c ^NS^ |
| TNF-α+IFN-γ | 0.02 | 0.01 | 0.02 | 0.001 | 0.0006 | 0.00003 | a ^NS^ b ^NS^ c^NS^ |
| Control | 0.0002 | 0.00005 | 0.0003 | 0.0001 | NA | NA | d ^NS^ |
| ***TGF-β1*** |  |  |  |  |  |  |  |
| TNF-α | 0.01 | 0.0008 | 0.02 | 0.001 | 0.004 | 0.00006 | a** b* c*** |
| IFN-γ | 0.0006 | 0.00002 | 0.001 | 0.0003 | 0.000003 | 0.000002 | a** b* c** |
| TNF-α+IFN-γ | 0.009 | 0.001 | 0.008 | 0.001 | 0.003 | 0.0001 | a ^NS^  b* c* |
| Control | 0.007 | 0.001 | 0.006 | 0.0007 | NA | NA | d ^NS^ |
| ***PTGER-2α*** |  |  |  |  |  |  |  |
| TNF-α | 0.003 | 0.001 | 0.005 | 0.002 | 0.0002 | 0.00009 | a ^NS^ b ^NS^ c ^NS^ |
| IFN-γ | 0.005 | 0.0005 | 0.0008 | 0.00005 | 0.003 | 0.0005 | a** b* c* |
| TNF-α+IFN-γ | 0.02 | 0.004 | 0.002 | 0.0004 | 0.003 | 0.0001 | a** b** c _NS_ |
| Control | 0.001 | 0.0002 | 0.001 | 0.0001 | NA | NA | d ^NS^ |
| ***HGF*** |  |  |  |  |  |  |  |
| TNF-α | 0.0002 | 0.0001 | 0.006 | 0.001 | 0.00002 | 0.000006 | a** b ^NS^ c** |
| IFN-γ | 0.0006 | 0.00002 | 0.001 | 0.0003 | 0.0000001 | 0.0000002 | a* b ^NS^ c** |
| TNF-α+IFN-γ | 0.001 | 0.0003 | 0.02 | 0.01 | 0.00003 | 0.00000001 | a* b ^NS^ c* |
| Control | 0.0001 | 0.00001 | 0.00002 | 0.000008 | NA | NA | d**** |
| ***VEGF*** |  |  |  |  |  |  |  |
| TNF-α | 0.01 | 0.001 | 0.1 | 0.004 | 0.04 | 0.002 | a**** b** c** |
| IFN-γ | 0.005 | 0.0001 | 0.08 | 0.003 | 0.006 | 0.0009 | a**** b ^NS^ c**** |
| TNF-α+IFN-γ | 0.02 | 0.001 | 0.1 | 0.03 | 0.003 | 0.0009 | a** b ^NS^ c** |
| Control | 0.005 | 0.0001 | 0.06 | 0.002 | NA | NA | d<**** |
| ***IL-8*** |  |  |  |  |  |  |  |
| TNF-α | 0.2 | 0.03 | 0.05 | 0.009 | 0.01 | 0.002 | a**** b**** c ^NS^ |
| IFN-γ | 0.01 | 0.002 | 0.0007 | 0.0002 | 0.001 | 0.0001 | a*** b*** c _NS_ |
| TNF-α+IFN-γ | 0.09 | 0.01 | 0.02 | 0.003 | 0.04 | 0.02 | a** b* c ^NS^ |
| Control | 0.002 | 0.0001 | 0.002 | 0.0001 | NA | NA | d ^NS^ |
| ***IL-1β*** |  |  |  |  |  |  |  |
| TNF-α | 0.005 | 0.001 | 0.0002 | 0.00005 | 0.003 | 0.001 | a* b ^NS^ c ^NS^ |
| IFN-γ | 0.004 | 0.0005 | 0.00002 | 0.00001 | 0.004 | 0.0002 | a*** b ^NS^ c*** |
| TNF-α+IFN-γ | 0.0008 | 0.0005 | 0.0001 | 0.00006 | 0.002 | 0.0002 | a ^NS^ b* c** |
| Control | 0.002 | 0.001 | 0.002 | 0.0003 | NA | NA | d ^NS^ |
