# Supplementary material for: Pluripotency and immunomodulatory signatures of canine induced pluripotent stem cell-derived mesenchymal stromal cells are similar to harvested mesenchymal stromal cells
Source: Sci Rep. 2021 Feb 10;11:3486. doi: 10.1038/s41598-021-82856-3 (PMC7875972; doi:10.1038/s41598-021-82856-3)
Supplement: Supplementary file 4 — Supplementary Table 3. [file 41598_2021_82856_MOESM4_ESM.docx]

Pluripotency and immunomodulatory signatures of canine induced pluripotent stem cell-derived mesenchymal stromal cells are similar to harvested mesenchymal stromal cells. Arash Shahsavari, Prasanna Weeratunga, Dmitry A. Ovchinnikov, and Deanne J. Whitworth.

**Supplementary Table 3.** **Effect of mitogen-stimulated canine lymphocytes on inflammatory cytokine expression of ciMSCs and cAT-MSCs.** (a) cAT-MSCs vs. co-cultured cAT-MSCs; (b) ciMSCs vs. co-cultured ciMSCs. Not significant (NS) p> 0.05; * p≤0.05; ** p≤0.005; *** p≤0.0002; **** p≤0.0001.

.

| Cytokine | cAT-MSCs | SEM | ciMSCs | SEM | Co-cultured  cAT-MSCs | SEM | Co-cultured  ciMSCs | SEM | *p value* |
| --- | --- | --- | --- | --- | --- | --- | --- | --- | --- |
| *iNOS* | 0.001 | 0.00007 | 0.005 | 0.0004 | 0.001 | 0.0002 | 0.002 | 0.0004 | a^NS^ b^****^ |
| *IDO* | 0.0005 | 0.00007 | 0.0004 | 0.00001 | 0.0002 | 0.00005 | 0.001 | 0.0001 | a ^NS^ b ^*^ |
| *GAL-9* | 0.002 | 0.0003 | 0.002 | 0.0004 | 0.007 | 0.001 | 0.001 | 0.0003 | a ^*^ b ^NS^ |
| *COX-2* | 0.0003 | 0.0001 | 0.0002 | 0.00005 | 0.0006 | 0.0002 | 0.001 | 0.0002 | a ^NS^ b^**^ |
| *TGF-β1* | 0.006 | 0.0007 | 0.007 | 0.001 | 0.0000001 | 1E-09 | 0.0004 | 0.0001 | a ^****^ b ^****^ |
| *PTGER-2α* | 0.001 | 0.0001 | 0.01 | 0.0002 | 0.002 | 0.0006 | 0.002 | 0.0003 | a ^NS^ b ^****^ |
| *HGF* | 0.00002 | 0.000008 | 0.0001 | 0.00001 | 0.00004 | 0.000005 | 0.00004 | 0.000007 | a ^NS^ b ^****^ |
| *VEGF* | 0.06 | 0.002 | 0.005 | 0.0001 | 0.008 | 0.002 | 0.01 | 0.002 | a ^****^ b ^NS^ |
| *IL-8* | 0.002 | 0.0001 | 0.002 | 0.0001 | 1.8 | 0.3 | 0.01 | 0.001 | a ^****^ b ^NS^ |
| *IL-1β* | 0.002 | 0.0003 | 0.002 | 0.001 | 0.02 | 0.004 | 0.03 | 0.003 | a ^*^ b ^**^ |
