# Supplementary material for: Pluripotency and immunomodulatory signatures of canine induced pluripotent stem cell-derived mesenchymal stromal cells are similar to harvested mesenchymal stromal cells
Source: Sci Rep. 2021 Feb 10;11:3486. doi: 10.1038/s41598-021-82856-3 (PMC7875972; doi:10.1038/s41598-021-82856-3)
Supplement: Supplementary file 5 — Supplementary Table 4. [file 41598_2021_82856_MOESM5_ESM.docx]

Pluripotency and immunomodulatory signatures of canine induced pluripotent stem cell-derived mesenchymal stromal cells are similar to harvested mesenchymal stromal cells. Arash Shahsavari, Prasanna Weeratunga, Dmitry A. Ovchinnikov, and Deanne J. Whitworth.

**Supplementary Table 4.** **Effect of cMSCs on inflammatory cytokine expression of mitogen-stimulated canine lymphocytes.** (a) Lymphocyte control vs. Co-cultured lymphocytes and cAT-MSCs; (b) Lymphocyte control vs. Co-cultured lymphocytes and ciMSCs; (c) Co-cultured lymphocytes with cAT-MSCs vs. Co-cultured lymphocytes with iMSCs. Not significant (NS) p> 0.05; * p≤0.05; ** p≤0.005; *** p≤0.0002; **** p≤0.0001.

| Cytokine | Lymphocyte  control | SEM | Co-cultured lymphocytes  & cAT-MSCs | SEM | Co-cultured lymphocytes  & ciMSCs | SEM | *P value* |
| --- | --- | --- | --- | --- | --- | --- | --- |
| *iNOS* | 0.001 | 0.00005 | 0.0007 | 0.00009 | 0.001 | 0.0007 | a ^NS^ b ^NS^ c ^NS^ |
| *IDO* | 0.0002 | 0.00001 | 0.0003 | 0.00002 | 0.0004 | 0.0001 | a ^NS^ b ^NS^ c ^NS^ |
| *GAL-9* | 0.2 | 0.005 | 0.008 | 0.002 | 0.001 | 0.0007 | a **** b ****  c ^NS^ |
| *COX-2* | 0.0008 | 0.0001 | 0.01 | 0.004 | 0.001 | 0.0008 | a ** b ^NS^ c ** |
| *TGF-β1* | 0.0002 | 0.0001 | 0.001 | 0.0001 | 0.0002 | 0.0001 | a *** b ^NS^ c *** |
| *PTGER-2α* | 0.008 | 0.0001 | 0.001 | 0.0004 | 0.001 | 0.0005 | a **** b ****  c ^NS^ |
| *HGF* | 0.00002 | 0.000002 | 0.00006 | 0.000007 | 0.00002 | 0.00001 | a * b ^NS^ c ^NS^ |
| *VEGF* | 0.03 | 0.001 | 0.009 | 0.002 | 0.01 | 0.006 | a * b * c ^NS^ |
| *IL-8* | 0.14 | 0.002 | 0.12 | 0.002 | 0.23 | 0.11 | a ^NS^ b ^NS^ c ^NS^ |
| *IL-1β* | 0.04 | 0.002 | 0.05 | 0.01 | 0.08 | 0.04 | a ^NS^ b ^NS^ c ^NS^ |
