# Supplementary material for: Pluripotency and immunomodulatory signatures of canine induced pluripotent stem cell-derived mesenchymal stromal cells are similar to harvested mesenchymal stromal cells
Source: Sci Rep. 2021 Feb 10;11:3486. doi: 10.1038/s41598-021-82856-3 (PMC7875972; doi:10.1038/s41598-021-82856-3)
Supplement: Supplementary file 6 — Supplementary Table 5. [file 41598_2021_82856_MOESM6_ESM.docx]

| ***p value*** | | | | |
| --- | --- | --- | --- | --- |
| **Treatment groups** | ***TGF-β1*** | ***VEGF*** | ***IL-8*** | ***IL-1β*** |
| Lymphocytes vs. cAT-MSCs only | ++++ | ++++ | ++++ | + |
| Lymphocytes vs. ciMSCs only | ++++ | ++++ | ++++ | ++ |
| Lymphocytes vs. Co-cultured Lymphocytes (with cAT-MSCs) | ns | + | +++ | ++++ |
| Lymphocytes vs. Co-cultured Lymphocytes (with ciMSCs) | +++ | ++++ | ++++ | ++++ |
| cAT-MSCs only vs. ciMSCs only | + | ++++ | ns | ns |
| cAT-MSCs only vs. Co-cultured Lymphocytes (with cAT-MSCs) | ++++ | ++++ | ++++ | +++ |
| cAT-MSCs only vs. Co-cultured Lymphocytes (with ciMSCs) | ns | ns | ++++ | + |
| ciMSCs only vs. Co-cultured Lymphocytes (with cAT-MSCs) | ++++ | ++++ | ++++ | ++ |
| ciMSCs only vs. Co-cultured Lymphocytes (with ciMSCs) | +++ | ++++ | ++++ | ns |
| Co-cultured Lymphocytes (with cAT-MSCs) vs. Co-cultured Lymphocytes (with ciMSCs) | +++ | ++++ | ++++ | ns |

Pluripotency and immunomodulatory signatures of canine induced pluripotent stem cell-derived mesenchymal stromal cells are similar to harvested mesenchymal stromal cells. Arash Shahsavari, Prasanna Weeratunga, Dmitry A. Ovchinnikov, and Deanne J. Whitworth.

**Supplementary Table 5.** **Effects of co-culture on the secretion of factors by lymphocytes and cMSCs.** A statistical comparison of the concentrations of Transforming growth factor-β1 (TGF-β1), Vascular endothelial growth factor (VEGF), Interleukin-8 (IL-8) and Interleukin-1β (IL-1β) as measured by ELISA in the supernatant collected from cultures of lymphocytes, ciMSCs and cAT-MSCs, and co-cultures of lymphocytes with each of the ciMSCs and cAT-MSCs. Significance is defined as: ns = not significant *p*>0.05; + *p*≤0.05; ++ *p*≤0.005; +++ *p*≤0.0002; ++++ *p*≤0.0001.
